# Another Consequence of the Warburg Effect? Metabolic Regulation of Na^+^/H^+^ Exchangers May Link Aerobic Glycolysis to Cell Growth

**DOI:** 10.3389/fonc.2020.01561

**Published:** 2020-08-18

**Authors:** Eivind Salmorin Birkeland, Lisa Maria Koch, Reinhard Dechant

**Affiliations:** ^1^Institute of Biochemistry, Department of Biology, ETH Zürich, Zurich, Switzerland; ^2^Life Science Zurich, Ph.D. Program for Molecular Life Sciences, Zurich, Switzerland

**Keywords:** Na^+^/H^+^-exchanger, cytosolic pH, growth and proliferation, metabolism, aerobic glycolysis

## Abstract

To adjust cell growth and proliferation to changing environmental conditions or developmental requirements, cells have evolved a remarkable network of signaling cascades that integrates cues from cellular metabolism, growth factor availability and a large variety of stresses. In these networks, cellular information flow is mostly mediated by posttranslational modifications, most notably phosphorylation, or signaling molecules such as GTPases. Yet, a large body of evidence also implicates cytosolic pH (pHc) as a highly conserved cellular signal driving cell growth and proliferation, suggesting that pH-dependent protonation of specific proteins also regulates cellular signaling. In mammalian cells, pHc is regulated by growth factor derived signals and responds to metabolic cues in response to glucose stimulation. Importantly, high pHc has also been identified as a hall mark of cancer, but mechanisms of pH regulation in cancer are only poorly understood. Here, we discuss potential mechanisms of pH regulation with emphasis on metabolic signals regulating pHc by Na^+^/H^+^-exchangers. We hypothesize that elevated NHE activity and pHc in cancer are a direct consequence of the metabolic adaptations in tumor cells including enhanced aerobic glycolysis, generally referred to as the Warburg effect. This hypothesis not only provides an explanation for the growth advantage conferred by a switch to aerobic glycolysis beyond providing precursors for accumulation of biomass, but also suggests that treatments targeting pH regulation as a potential anti-cancer therapy may effectively target the result of altered tumor cell metabolism.

## pHc Regulates Cell Growth and Proliferation

**FIGURE 1:**
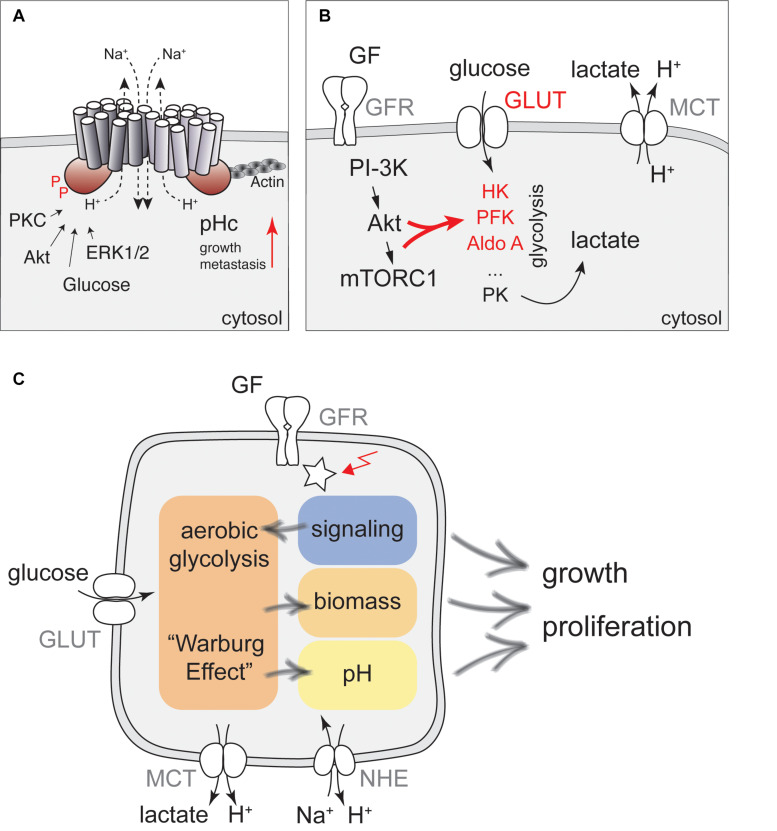
A network of cellular metabolism and cellular signaling governs cell growth and regulation of pHc. **(A)** NHE1 is a key regulator of pHc in mammalian cells. A schematic representation of the NHE1 structure together with key regulatory inputs and potential functions for cancer development is shown. **(B)** Regulation of glycolytic activity by PI3K/Akt signaling. Glucose transporters (GLUT) and the metabolic enzymes hexokinase (HK), phosphofructokinase (PFK), aldolase A (Aldo A) indicated in red are all direct or indirect targets of growth factor (GF) dependent regulation, leading to enhanced aerobic glycolysis upon stimulation with GFs. Glycolytic activity is also directly coupled to pHc regulation by lactate export using Monocarboxylate transporters. **(C)** Proposed model for how signaling, metabolism, and pH interact to regulate cell growth and proliferation. Glucose metabolism can be stimulated by glucose, growth factors and oncogenic activation (red arrow) to produce precursors for biomass production, but may also increase pHc through activation of NHE activity, further contributing to enhanced cell growth and proliferation.

Evidence for pH-dependent cell growth is largely based on experiments modulating the activity of Na^+^/H^+^-exchangers (NHE) of the SLC9A family of transport proteins. These proteins regulate intracellular pH by using the Na^+^ gradient to transport protons across their target membranes. NHE1-5 (SLC9A1-5) localize to the plasma-membrane and regulate pHc (1). In contrast, NHE6-9 are found in organelles of the endomembrane system to regulate luminal pH, but can also affect pHc (1, 2). NHE proteins form homo-dimers with 12 transmembrane domains located in the N-terminal part of the protein and a large C-terminal cytoplasmic domain, which is target of several kinases. The C-terminal domain also interacts with phospholipids and the actin cytoskeleton to regulate cell migration and contribute to metastasis [Figure 1A and Ref. (3)].

In addition to NHEs, several other regulators of pHc have been identified. These include Na^+^/Bicarbonate transporters and monocarboxylate transporters (MCTs), ATP-driven proton pumps, as well as carbonic anhydrases (4). All these pH regulators have been linked to a growing number of physiological activities including regulation of cellular signaling, transcription and cell growth, and have been associated with cancer (5–9). While NHE proteins are ubiquitously expressed to regulate intracellular pH (10), other pH regulators become critical only under specific conditions. For example, MCTs, which transport lactate and protons across the plasma-membrane, are critical to maintain pHc in rapidly proliferating cells and primary tumors, but are less important in differentiated cells (11–14).

A large body of evidence suggests that activation of NHE proteins upon growth factor stimulation is a critical step in promoting cell growth and proliferation (15). Growth factor stimulation triggers an increase in pHc of about 0.2–0.3 pH units (16–24). As this increase in pH is tightly correlated with increased Na^+^ influx and blocked by amiloride, it was concluded that pH regulation depends on Na^+^/H^+^-exchangers. Similar conclusions were drawn from overexpression or injection of activated Ras into quiescent cells (25, 26), or overexpression of v-Mos (26). Importantly, increased pHc is also necessary for initiation of cell-cycle progression under these conditions (25, 27). Although the increase in pHc is maximal several minutes following injection of active Ras, addition of amiloride as late as 6 h after the injection of the activated protein effectively suppresses DNA replication (25), suggesting that high pHc may act at a specific step during early G1. Interestingly, increased pHc in response to growth factor stimulation is also necessary for high translation efficiency and correlates with phosphorylation of the ribosomal protein S6 (28), a critical target of the mTORC1 pathway (29). mTORC1 activity was subsequently confirmed to be sensitive to pH (30) and inhibition of NHE activity (31). The underlying mechanisms remain to be fully established, but may involve regulation of amino-acid uptake by macropinocytosis (31).

In addition, elevated pHc may further promote cell growth by regulating metabolism through the intrinsic pH-sensitivity of metabolic enzymes. In particular, phosphofructokinase and lactate dehydrogenase have received special attention due to a sharp pH optimum at slightly alkaline pH, which may lead to enhanced glycolysis and glucose uptake upon NHE activation (32–34).

Increased NHE activity has also been linked to multiple aspects of cancer and causes a reversed proton gradient across the plasma-membrane due to elevated pHc, enhanced tumor growth and invasion (3, 35–38). Accordingly, pharmacological inhibitors of NHE activity have been proposed as anti-cancer therapies (39–42). Indeed, the FDA approved drug amiloride, a broad specificity NHE inhibitor and potassium sparing diuretic, or derivatives thereof, are highly effective in mouse models for multiple myeloma (43) and pancreatic cancer (2). High NHE expression correlates with poor survival in several cancer types, further underscoring the importance of pH regulation in these diseases (2, 44). Mechanisms for increasing NHE activity may vary in different cancers and might include increasing the specific activity of NHE proteins. Therefore, detailed understanding of NHE regulation is needed to better understand their contribution to the disease.

## Regulation of NHE Activity by Mitogenic Kinases

Regulation of NHE activity in response mitogenic stimuli has been mostly attributed to direct phosphorylation of NHE proteins by growth factor activated kinases. Indeed, activation of Protein Kinase C (PKC) by phorbol esters (15–17, 45–47), or its endogenous activator diacyl-glyceride triggers an NHE-dependent increase in pHc (16, 48, 49), while inhibition of PKC by trifluoperazine can abolish the rise in pHc upon growth factor stimulation (50). Thus, it was suggested that PKC is the primary target downstream of growth factors for pH regulation. Yet, as no PKC-dependent phosphorylation sites on NHE proteins have been identified, the effect of PKC on NHE activation may be indirect.

In contrast, both Akt and Erk1/2 have been suggested to directly phosphorylate NHE1 (Figure 1A). *In vitro*, Akt phosphorylates NHE1 at Ser-648, while Erk1/2 phosphorylates NHE1 at Ser-770 and Ser-771 (51–55). Akt is required to reestablish physiological pH following an acidification stress due to acid loading and mutating S648 to a non-phosphorylatable residue impairs NHE1 function (52), strongly suggesting that Akt-dependent phosphorylation of NHE1 is key for increased pHc and cell proliferation. Yet, evidence for *in vivo* phosphorylation of this site has remained limiting. Nevertheless, these phosphorylation sites are conserved in NHE2 and NHE4, and other NHE proteins harbor MAPK or CDK consensus sites (SP) at the corresponding position. Thus, phosphorylation might be a general mechanism for the regulation of Na^+^/H^+^-exchangers.

Excellent reviews are available that summarize NHE regulation by phosphorylation (10, 51, 53). Here, we rather focus on the interaction between cellular metabolism and pHc. We hypothesize that an increased pHc and increased glycolytic activity, both commonly found in cancers, are two sides of the same coin, which contribute to enhanced cell growth and proliferation. Specifically, we discuss potential mechanisms of regulation of Na^+^/H^+^-exchange by cellular metabolism as a mechanism to control cell growth.

## Aerobic Glycolysis May Increase pHc via NHE1 Activation

Several reports have shown that glucose stimulation of cells is sufficient to increase pHc in an NHE1-dependent manner. Specifically, glucose availability stimulates pHc via NHEs in pancreatic beta-cells, which may contribute to glucose stimulated insulin release (56–60). NHE-dependent regulation of pHc by glucose was also observed in liver or muscle cells (61–63), but the underlying mechanisms are only poorly understood. Importantly, glucose-dependent regulation of pHc can be observed in the absence of growth-factors (59, 60), and inhibition of glycolysis by 2-deoxy-glucose strongly decreased NHE activity (64–66), suggesting that Na^+^/H^+^-exchange is regulated by a metabolic signal derived from glycolysis, or might be coupled to energy metabolism.

Increased glucose uptake and metabolism to fuel aerobic glycolysis is also a general feature of mitogenic stimulation under physiological conditions and oncogenic transformation [Figure 1B and Refs. (67–69)] and is generally referred to as the Warburg effect. Although it was originally assumed that tumor cells upregulate glycolysis even in the presence of oxygen due to defective mitochondria, it is now clear that increased rates of glycolysis allow the redirection of metabolic fluxes toward more efficient biomass production (69, 70) in actively proliferating cells and form the molecular basis of using PET scans for tumor detection (71).

However, if NHE activity is regulated by a metabolic signal in response to increased glucose concentration, the same mechanisms activating NHE activity should also be in place when glucose uptake and glycolysis are activated by growth factors or oncogenic transformation. Thus, understanding the regulation of NHE activity by metabolic cues might identify mechanisms how altered tumor cell metabolism contributes to elevated pHc and enhanced cell growth (Figure 1C).

Activation of aerobic glycolysis is best understood in response to PI3-kinase signaling and Akt activation, which has been suggested to be the Warburg kinase (72) (Figure 1B). Akt is required for growth factor-stimulated glucose uptake by triggering translocation of glucose transporters (GLUTs) to the plasma-membrane (73–75), via phosphorylation of the GTPase activating protein (GAP) for Rab10, AS160 (76). mTORC1 activation downstream of PI3-kinase/Akt signaling also promotes expression of Hexokinase II (77–79). In addition, Akt phosphorylates Hexokinase to promote its association with mitochondria, which protects cells from apoptosis, but may not contribute to enhanced enzymatic activity (80). Akt also indirectly stimulates phosphofructokinase 1 (PFK-1), the major control point of glycolysis. Specifically, Akt promotes the accumulation of the glycolytic side-product and most potent allosteric activator of PFK-1, Fructose-2,6-bisphosphate by phosphorylation of PFK-2 (81, 82). Finally, PI3-kinase signaling increases Aldolase A activity (83). Collectively, these processes lead to enhanced glycolytic activity and ATP production (84).

The PI3-Kinase/Akt pathway is probably the most frequently activated pathway in cancer, therefore explaining the shift toward high aerobic glycolysis in a large number of tumors. However, at least on a transcriptional level, upregulation of glucose uptake and glycolysis can also be observed upon activation of c-myc, mTORC1, and K-Ras, or upon loss of p53 (85–91), further underscoring the importance of enhanced aerobic glycolysis to sustain, or even signal enhanced cell growth.

## Potential Mechanisms of NHE Regulation by Glucose Metabolism

**FIGURE 2:**
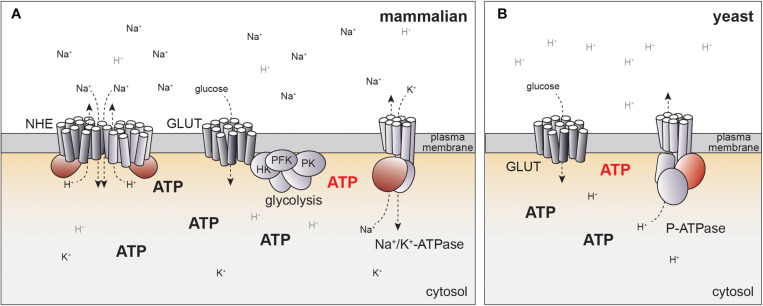
Potential mechanisms for how NHE activity can be linked to glucose metabolism. **(A)** In mammalian cells, pHc is regulated by NHE activity, which may be linked to ATP production directly or indirectly via Na^+^/K^+^-ATPase. Localized production of ATP by glycolysis at the plasma membrane may generate distinct pools of ATP (red) that might help to explain coupling of cellular metabolism to pH regulation. Potential regulatory or catalytic ATP binding sites in ion pumps and exchangers are indicated in red. See text for details. **(B)** Evolutionary conservation of glucose-dependent regulation of pHc. In yeast, pHc is regulated by an ATP-dependent proton pump (P-ATPase) independent of Na^+^/H^+^ exchange. P-ATPase activity is coupled to glycolytic activity, but molecular mechanisms remain to be fully established. Color code for ATP regulated domains and hypothetical localized ATP pools same as in panel **(A)**.

How could glucose metabolism be linked to NHE-dependent pH regulation? In principle, glucose could regulate the specific activity of NHE proteins, or affect the Na^+^ gradient by the Na^+^/K^+^-ATPase (Figure 2A). Similar to NHE proteins, the Na^+^/K^+^-ATPase is also subject to growth factor-dependent activation and the same kinases regulating NHEs have been linked to activation of the Na^+^/K^+^-ATPase (92–94). In particular, PKC directly phosphorylates Na^+^/K^+^-ATPase, possibly explaining PKC-dependent activation of Na^+^/H^+^-exchange (95). Indeed, Na^+^/K^+^-ATPase activity rapidly increases upon glucose stimulation in different cell types (96, 97), which could translate into changes of pHc.

At least two key kinases regulating NHEs respond to changes in cellular metabolism. PKC is regulated by glucose availability through modulating the levels of its activator DAG (98). Similarly, Erk1/2 activity is subject to glucose regulation (99, 100). Thus, both kinases could directly or indirectly contribute to NHE activation in response to glucose (101). Yet, careful assessment of the basal activity of these kinases and NHE phosphorylation in the absence of growth factors would be required to study a potential role in glucose-dependent regulation of pHc, before potential molecular mechanisms can be addressed.

### Direct Regulation of NHE Activity by Metabolic Cues

Conceptually, direct coupling of metabolism to NHE activity may be more appealing. In particular, coupling of glycolytic activity, or flux, to pH regulation would readily explain the observed increase in pHc by glucose availability, stimulation of glucose metabolism by mitogenic activation, and metabolic reprogramming in cancer. Interestingly, in all highly glycolytic cells, glycolysis is directly coupled to pHc via MCTs, which secrete lactic acid, the end-product of fermentation (102, 103). This is also the basis of using extracellular acidification rates as a means of estimating glycolytic flux (104, 105). Yet, it is less clear how glycolysis could be coupled to NHE activity.

It has been hypothesized that sensing of metabolic flux may depend on the accumulation of metabolites, which tightly correlate with pathway activity and trigger the appropriate cellular response (106). For example, the abundance of fructose-1,6-bisphosphate (FBP) tightly correlates with glycolytic flux in yeast and bacteria, and binding of FBP to a transcription factor allows for coupling of glycolytic flux to gene expression (107, 108). Similarly, in pancreatic beta-cells, ATP correlates with glucose concentration and glycolytic activity and triggers insulin secretion by binding to ATP-sensitive K^+^-channels (109).

ATP concentration may also link glycolytic flux to the regulation of pHc by NHE activity. As discussed above, Akt activation results in increased ATP concentrations (84). Moreover, the establishment of the Na^+^ gradient necessary for NHE activity consumes a large fraction of cellular ATP production (110), suggesting tight linkage to energy metabolism.

Indeed, NHE1 activity directly depends on the presence of ATP. Basal Na^+^/H^+^-exchange can occur in the absence of ATP based on the concentration gradient of the transported cations (111, 112). Yet, depletion of cells from ATP abolishes NHE activity (65, 66), while readdition of ATP restores NHE activity in patch-clamp experiments (65). Half maximal activation of NHE1 was achieved at 5 mM ATP, suggesting that NHE activity could be modulated by changes in the ATP concentration *in vivo*. Interestingly, ATP depletion reduces the affinity of NHE proteins to protons by 0.5 pH units, readily explaining ATP-dependent pH regulation (66, 113).

Surprisingly, NHE activity could also be triggered by the poorly hydrolysable ATP analog ATPγS and did not depend on the presence of Mg^2+^ (65). Thus, ATP-dependency is unlikely to be mediated by associated kinases or ATPases. Instead, Na^+^/H^+^-exchange may depend on direct binding of ATP to NHE proteins or a membrane associated activator. Consistently, ATP dependence of NHE1 requires the presence of its cytoplasmic C-terminal domain (114, 115). Although no consensus sequences from known ATP-binding motives can be identified in the primary sequence, cross-linking experiments have revealed evidence for direct binding of ATP to the cytoplasmic domain of NHE1 (116). While mapping of the potential ATP binding site will be required to generate mutants to directly test the significance of ATP binding *in vivo*, this model offers an attractive mechanism of coupling metabolic activity to NHE activity (Figure 2A).

### Indirect Regulation of NHE Activity by Metabolic Cues

An alternative model for coupling of energy metabolism to regulation of pHc through ATP-dependent ion pumps may be suggested by evolutionary considerations of pH-dependent cell growth. In yeast, pHc is regulated by a P-type ATPase, PMA1, that directly pumps protons across the plasma-membrane in an ATP-dependent manner (117, 118), but does not require NHE activity at the plasma-membrane (Figure 2B). PMA1 activity and pHc increase with the quality and quantity of the available carbon source (119). As in mammalian cells, high pHc drives cell growth and proliferation, at least in part, by activating TORC1 (99, 119).

The differences in pH regulation in yeast and mammals are readily explained by the different environmental constraints for single cellular organisms and cells embedded within a complex organism. In their natural environment yeast cells are constantly exposed to changes in osmolarity and thus may rely on ATP-dependent proton pumps rather than a Na^+^ gradient. In contrast, establishing a proton gradient with a similar concentration profile as the Na^+^ gradient in mammalian cells (120, 121) would yield pH differences of more than 1 pH unit across the plasma-membrane and may thus require indirect regulation of pH via Na^+^-H^+^-exchange. Yet, the conservation of pHc as a glucose-dependent signal regulating cell growth indicates that regulation of pH in mammalian cells may also be mediated by coupling of Na^+^/K^+^-ATPase to glucose metabolism (Figure 2A).

Indeed, several ATP-dependent ion pumps have been suggested to be regulated by energy metabolism in mammalian cells. In particular, direct modulation by ATP has been proposed for ATP-dependent cation exchangers and ion channels (122–130) despite the fact that the Km of these pumps for ATP hydrolysis have been consistently found to be significantly lower than physiological ATP concentrations (131–133), making a direct coupling of their activity to changing ATP concentrations in cells unlikely. To resolve this contradiction, it has been suggested that these pumps may be coupled to glycolytic ATP production by concentrating ATP producing enzymes close to the ATP-dependent pumps, thereby allowing physical or kinetic coupling of ATP synthesis to hydrolysis (124, 134). For example, glycolytic enzymes including hexokinase, phosphofructokinase and pyruvate kinase co-purify with the plasma-membrane in pancreatic cancer cells, which may allow direct regulation of an ATP-dependent Ca^2+^-pump (124). In cardiomyocytes and erythrocytes, glycolytic enzymes localize at the plasma-membrane (130, 135) and have been suggested to regulate Na^+^/K^+^-ATPase (122) as well as ATP-sensitive K^+^-channels (130, 134), possibly by direct interaction and localized ATP production (125).

Theoretical considerations argue against the formation of localized pools of metabolites based on local enrichment of metabolic enzymes, as rapid intracellular diffusion of metabolites outcompetes even the fastest enzymes, leading to rapid dissipation of local concentration differences (136). Yet, it remains a possibility that *in vitro* determination of enzymatic parameters fall short of accurately replicating the specific conditions in the microenvironment at the target membranes. Interestingly, indirect measurements of local ATP concentrations by targeting luciferase, which emits light in an ATP-dependent manner, to different cellular locations clearly support the existence of separated, local pools of ATP. Specifically, this method allows to follow the dynamics of ATP concentration in different cellular compartments following a glucose pulse. While ATP only very transiently accumulates in the cytosol upon glucose stimulation, ATP stabilizes at elevated levels at the plasma-membrane upon glucose stimulation (137). More detailed measurements of localized metabolite distributions, for example using FRET reporters would be key to further support localized pools of metabolites regulating ATP-dependent processes at the plasma-membrane. Similarly, to discriminate between direct and indirect mechanisms to couple metabolism to NHE activity, dynamic measurements of Na^+^/K^+^-ATPase and/or the resulting Na^+^ gradient would be required.

## Conclusion

While the molecular mechanisms linking glucose or energy metabolism to increased pHc await further clarification, the existing data strongly argue for a tight coupling of glucose metabolism to NHE activity. As glucose metabolism can be stimulated by increasing glucose concentration or induction of the Warburg effect, this coupling readily explains the elevated pHc found in cancer cells. In turn, an elevated pHc may further enhance aerobic glycolysis due to the pH-sensitivity of phosphofructokinase (33) as part of a positive feedback loop to regulate cell growth. Thus, an elevated pHc may act as a signal that relays changes in cellular metabolism to cell growth and proliferation, but further identification of pH-sensitive steps governing cell growth and proliferation will be required to better understand the functional importance of pHc in normal and cancer cells.

Targeting cellular metabolism for cancer therapy has triggered great interest as potential treatments could be widely applicable to a range of tumors with similar metabolic alterations. Indeed, several glycolytic inhibitors have been tested as potential anti-cancer treatments, but have largely failed due to high toxicity at the effective dose (138, 139). Targeting pH regulation by NHE proteins may act as a treatment of the consequences of altered tumor metabolism and may help to devise novel treatment strategies. While similar considerations of toxicity by targeting NHE proteins may apply, a better understanding of NHE regulation in normal and cancer cells will help to further dissect the interplay between cellular metabolism and signaling, and to define windows of opportunities to treat cancer by targeting pHc regulation.

## Author Contributions

All authors listed have made a substantial, direct and intellectual contribution to the work, and approved it for publication.

## Conflict of Interest

The authors declare that the research was conducted in the absence of any commercial or financial relationships that could be construed as a potential conflict of interest.
